# Core Competencies for Medical Teachers (KLM) – A Position Paper of the GMA Committee on Personal and Organizational Development in Teaching

**DOI:** 10.3205/zma000965

**Published:** 2015-05-13

**Authors:** Anja Görlitz, Thomas Ebert, Daniel Bauer, Matthäus Grasl, Matthias Hofer, Maria Lammerding-Köppel, Götz Fabry, GMA Ausschuss Personal- und Organisationsentwicklung in der Lehre

**Affiliations:** 1 Klinikum der Universität München, Institut für Didaktik und Ausbildungsforschung in der Medizin, München, Deutschland; 2 Johann Wolfgang Goethe-Universität Frankfurt, Fachbereich Medizin, Frankfurter Arbeitsstelle für Medizindidaktik, Frankfurt, Deutschland; 3 Medizinische Universität Wien, Universitätsklinik für Hals-Nasen-Ohrenkrankheiten, Wien, Österreich; 4 Heinrich-Heine-Universität Düsseldorf, Studiendekanat Medizin, Arbeitsgruppe Medizindidaktik, Düsseldorf, Deutschland; 5 Eberhard-Karls-Universität Tübingen, Kompetenzzentrum für Hochschuldidaktik Medizin Baden-Württemberg, Tübingen, Deutschland; 6 Albert-Ludwigs-Universität Freiburg, Medizinische Psychologie und Soziologie, Freiburg, Deutschland

**Keywords:** faculty development, organizational development, teaching skills, teaching competencies, teach-the-teacher, higher education pedagogy

## Abstract

Recent developments in medical education have created increasing challenges for medical teachers which is why the majority of German medical schools already offer educational and instructional skills trainings for their teaching staff. However, to date no framework for educational core competencies for medical teachers exists that might serve as guidance for the qualification of the teaching faculty.

Against the background of the discussion about competency based medical education and based upon the international literature, the GMA Committee for Faculty and Organizational Development in Teaching developed a model of core teaching competencies for medical teachers. This framework is designed not only to provide guidance with regard to individual qualification profiles but also to support further advancement of the content, training formats and evaluation of faculty development initiatives and thus, to establish uniform quality criteria for such initiatives in German-speaking medical schools.

The model comprises a framework of six competency fields, subdivided into competency components and learning objectives. Additional examples of their use in medical teaching scenarios illustrate and clarify each specific teaching competency.

The model has been designed for routine application in medical schools and is thought to be complemented consecutively by additional competencies for teachers with special duties and responsibilities in a future step.

## Zusammenfassung

Die Entwicklungen in der Medizinischen Ausbildung der letzten Jahre konfrontieren Lehrende zunehmend mit neuen didaktischen Herausforderungen. An zahlreichen Standorten im deutschsprachigen Raum werden bereits Qualifizierungsangebote für Lehrende angeboten, jedoch fehlt bisher ein Orientierungsrahmen für medizindidaktische Kompetenzen, der ein Qualifikationsprofil für Lehrende darstellt.

Vor dem Hintergrund der Diskussion um die Kompetenzorientierung des Medizinstudiums und auf Grundlage aktueller internationaler Literatur wurde durch den GMA Ausschuss für Personal- und Organisationsentwicklung in der Lehre ein Kernkompetenzmodell für Lehrende in der Medizin entwickelt. Das Modell soll nicht nur den Lehrenden Orientierung zu ihrem Qualifikationsprofil geben, sondern auch die inhaltliche Ausrichtung hochschuldidaktischer (Aus-) Weiter- und Fortbildungen sowie die Evaluation von Fakultätsentwicklungsprozessen erleichtern und nicht zuletzt einheitliche Kriterien für die Beurteilung der Lehrqualifikation in deutschsprachigen Raum definieren.

Das Modell besteht aus sechs Kompetenzfeldern, für die jeweils Teilkompetenzen definiert und Lernziele beschrieben wurden. Anwendungsbeispiele sollen die jeweiligen Kompetenzen verdeutlichen.

Das Modell ist für die praktische Anwendung konzipiert und soll in einem nächsten Schritt durch spezifische Kompetenzen für Lehrende mit besonderen Aufgaben ergänzt werden.

## Background

This article introduces a model for core teaching competencies for medical teachers, which draws upon many sources:

An increasing number of research studies in medical education have elaborated well-grounded evidence about how to master the specific educational challenges in medical curricula. Thus, medical schools show an increasing awareness that medical teachers do not only have to deliver knowledge, but are at the same time confronted with new challenges: The teachers do not only have to master a broad spectrum of different teaching and assessment formats, but are also expected to contribute significantly to the improvement of their local curricula, provide teaching and learning materials and to reconcile teaching strategies and course content with colleagues from other disciplines. In addition, they have to secure an adequate level of evaluation and quality assurance. These multifaceted activities require a variety of specific competencies, which cannot be taken for granted. In this context, most German-speaking medical schools have established opportunities for their teaching personnel to gain at least basic or core educational skills [5]. However, a comprehensive framework of educational competencies outlining the qualification profile for medical educators was missing in German-speaking countries so far.

Education in general is changing towards a competency based approach: The international debate on the quality of medical education is witnessing a growing awareness that the successful acquisition of competencies which enable interns and residents to solve typical problems in patient care should be a major goal of UGME curricula. Therefore, in Anglo-Amercian countries several competency frameworks have been developed, of which the model proposed by the Royal College of Physicians and Surgeons in Canada (CanMEDS) [6] attracted the most attention internationally. Although this model has initially been developed for PGME, it is also increasingly applied to UGME-issues and has had a significant impact on discussion about the reform of medical education in German-speaking countries. For example, the CanMEDS model forms the basis of the second edition of the “Swiss Catalogue of Learning Objectives for Undergraduate Medical Training“ [2][, [3] and has also been chosen as a source for the German Competency-Based Catalogue of Learning Objectives in Medicine (NKLM) [7], [http://www.nklm.de accessed on 15.09.2014] which is currently under development. Thus, there will be a framework available in the near future, which will define the objectives of UGME in Germany consistently as competencies. The resulting challenges to future medical curricula will be complex and far-reaching, because the NKLM will not be a simple laundry list of objectives, but will also require different educational and methodological approaches that will live up to the competency orientation. This in turn will change the requirements for medical teachers.

## What are Competencies?

One of the biggest challenges in the discussion on competency-based curricula is the definition of the concept of “competency” itself. For a number of years “competency” has been a buzzword in educational and social sciences [9], but has also evolved to being an important key concept in the political realm since the mid 90s [1]. Most definitions of competence share the following four characteristics [8]:

 Competencies…

- … manifest themselves in the accomplishment of situational acts
- … refer to a specific situation or context
- … are linked to an individual
- … can be learned and modified

In this respect, competencies can be defined as

“[…] the available or learnable cognitive capabilities and skills of an individual to solve specific problems accompanied by the motivational, volitional and social dispositions and abilities to use the problem solutions in various situations successfully and responsibly” [14].

This definition also informs the NKLM but was supplemented by another definition that is more specific for medical practice:

“[…] Professional competence is the habitual and judicious use of communication, knowledge, technical skills, clinical reasoning, emotions, values, and reflections in daily practice for the benefit of the individual and community being served.” [4]

Although this definition has been devised for medical practice, it also makes sense with regard to medical teaching, because in both fields it is not sufficient to just apply pre-defined solutions but it is necessary instead to find individual solutions for, at times, complex and ill-defined problems. In medical education as in medical practice alike this requires the integration of knowledge, skills, and attitudes as well as emotional and value related aspects. Reflection is of utmost importance to this end, to adjust one's own action to the respective situational demands in a flexible way and to learn from the experiences just gained.

## Objectives

Against this background, the Committee on Personal and Organizational Development in Teaching (POiL) of the German Society for Medical Education (GMA), in close cooperation with the Network on Medical Education (MDN), a working group of the association of German medical schools (“Medizinischer Fakultätentag, MFT”) agreed to develop a framework of competencies, which encompasses those core competencies that are crucial for all medical teachers. As the term “core competencies” illustrates, the presented model describes basic competencies, which are independent of the individual teacher’s specific position, duties and responsibility.

The presented framework of competencies predominantly pursues pragmatic goals, e.g.

- to provide a guide for medical teachers, which specific competencies are expected from them,
- to serve as guidance for the conceptual design of faculty development initiatives related to teaching,
- to facilitate the evaluation of organizational development at the medial schools,
- to define consistent criteria for the assessment of medical teaching qualification in German-speaking countries, and
- to serve as a foundation and useful tool for further scientific research on learning processes in UGME as well as PGME.

## The development process

The model described here has been developed during an extensive consensus process under leadership of the POiL committee to achieve as much acceptance as possible among the German-speaking medical schools. The process embraced seven iterative steps in which the recommendations that were worked out by the respective working group of the POiL committee were repeatedly discussed by a more widespread group of experts from the German-speaking countries. The expert group brought together educators from medical and health professions education with considerable experience and scholarship in faculty and organizational development regarding educational issues in medicine and health care.

During a workshop of the POiL committee at the 2011 GMA conference in Munich the North American model worked out by Srinivasan et al. [12] was discussed and chosen as the starting point for the development of an own competency framework. During translation of the document it became clear that it was not possible to transfer the definition of the competencies in a one-on-one fashion to the German language and respective context of medical education and that an adaptation of the competencies was needed. Table 1 (Tab. 1) illustrates the further steps of the process. In October 2014 the finalized manuscript was submitted to the executive committee of the GMA.

## Results

The model of the core competencies for medical teachers (KLM) defines the following six competency fields:

- Educational action in medicine
- Learner centeredness
- Social and communicative competencies
- Role modelling and professionalism
- Reflection and advancement of personal teaching practice
- Systems related teaching and learning

These six competency fields are equally relevant. For each competency field competency components have been defined that are specified by learning objectives and illustrated by examples to facilitate their transfer into practice (see table 2 (Tab. 2)).

The KLM embraces six competency fields with 21 competency components that are operationalized by 57 learning objectives. These have been illustrated by 63 examples (see table 3 (Tab. 3)). To demonstrate this, table 4 (Tab. 4) presents an excerpt from the competency field “social and communicative competencies”.

The complete model “core competencies for medical teachers” with all competency fields, competence components, learning objectives and examples is enclosed in the attachment .

## Discussion and Outlook

The model of the KLM was based on the „Six Core Competencies for Medical Educators” as developed by Srinivasan et al. [12]. While the original partitioning into six competency fields has been preserved, the naming and further breakdown into competence components and learning objectives was adapted to the needs and conditions of teaching at medical schools in the German-speaking countries by means of an incremental consensus process. The unequal scope of the different competence fields is a result of this discussion process and does not necessarily reflect differences in relevance. On the other hand, some of the competence components and learning objectives could have been assigned to more than one competence field. The competence component “competent medical educators adequately assess and evaluate the learning progress of their students with regard to knowledge, skills and attitudes” for instance has been assigned to the competence field “Educational Action in Medicine” while it could also have been assigned to “Learner Centeredness”. Similar to what has been encountered during the development of the NKLM [7] [http://www.nklm.de accessed on 15.09.2014], finding the right granularity for competence components and learning objectives was challenging. As many individuals participated in the consensus process, harmonizing language and granularity was a primary concern in finalizing the model. Furthermore, some of the competence components were given different weightings by different individuals. Thus, the final version is the result of a consensus process where the usefulness of the product was considered more important than conceptual rigor.

Compared to other recommendations regarding competency requirements in (higher) education the current KLM model which embraces 21 competency components is less comprehensive, which will facilitates its use in practice. Nevertheless, only a few competencies and learning objectives that are enlisted in other models are actually missing. The comparison of the KLM with the “Core competencies in teaching and training for doctors in Scotland” [10] and the “Framework Areas for the Professional Development of Postgraduate Medical Supervisors” published by the Academy of Medical Educators [1] revealed that the KLM misses only three of the quite elaborate 80 “Core Competencies”. These are C46 (“Effectively appraise medical students, trainees, colleagues and members of the wider healthcare team”), C48 (“Adapt their own practice where benefits of using technology have been identified”) und C72 (“Demonstrate a standard of professional and educational practice consistent with the requirements of the General Medical Council”). These differences were discussed but not adjusted as the KLM only defines core competencies that are important for all medical teachers irrespective of their actual field of duty. C72 could not be integrated into KLM as no equivalent standard from a professional corporation in Germany exists and the medical licensing act does not define qualitative requirements for medical teachers. The competence field relating to the use of new media and technological innovations is not explicitly mentioned in the KLM. During the consensus process it was agreed that using new media is included in other competency components and learning objectives, e.g. “competent medical educators realize these competence components (i.e. …are able to design conducive teaching and learning processes with regard to methodological and educational issues) by analysing and creating learning processes adequately with regard to the surrounding conditions and by applying suitable methods and media”. Applying new media in teaching requires medical teachers to acquire the respective competencies. At first, such more specific competencies were excluded from the development of the KLM as the focus was more on comprehensive competencies that are essential for different teaching methods and formats. Definitions of more specific competencies will be published in a follow-up paper of our POiL working group. Compared to the five content areas of the “Certificate for Teaching in Higher Education of the Bavarian Universities” (Concepts for Learning and Teaching, Presentation and Communication, Assessment, Teaching as a Profession, Mentoring and Counselling) [http://www.profilehreplus.de/index.php?id=44 accessed on 08.08.2014] the competencies, competency components and learning objectives of the KLM are much more specific and related to teaching and learning in medicine (e.g. consideration of patients, specific teaching formats i.e. bedside teaching). This illustrates why it makes sense to develop specific pedagogical content knowledge and why it is reasonable to prepare teachers specifically for these teaching formats and requirements.

The competencies defined in the KLM relate to the pedagogical education and qualification of postgraduates but the pedagogical training should already start during medical school [13]. The current (9/2014) definition of competency components relating to the role of the “Scholar” (“Gelehrter”) in the NKLM emphasizes this claim. Another competency defined in the NKLM, “as a lifelong learner the graduate improves and maintains professional action through continuous learning”, stresses yet another aspect that is also taken up in the KLM as the competency for continuing professional development and lifelong learning. The demand for lifelong learning beyond what is defined with regard to the respective medical specialties in the regulations of the professional bodies is also a crucial aspect for teaching competency. While many medical faculties established structured programs for educational qualification that are a requirement for promotion and tenure (“Habilitation”) there is no consensus for medical teachers beyond that. This fact certainly deserves critical discussion especially with regard to the continuous development of learning and teaching that results in ever growing challenges that medical educators must master.

It is also necessary to compare the KLM model with the competencies that are defined for medical students in the NKLM within the role of the Scholar, as soon as this document is approved. This would allow the mapping of competencies that students might already acquire during their studies and would also help educators in charge of faculty development initiatives to recognize where students should already be included. Training for student tutors which is already systematically implemented at many medical schools [http://www.profil.uni-muenchen.de/tutorplus/ausbildung/index.html accessed on 19.09.2014] could then also be included in faculty development initiatives for teaching.

The KLM is a pragmatic model that can be used to map the faculty development initiatives for teaching at different sites. Testing the applied model is currently under way at a number of sites that offer educational qualification programs and write structured self-reports for the quality management process of the MDN. The KLM should provide guidance for that process. In this way it will be ascertained whether it will be possible to map the different programs by means of the KLM. If the model proves useful as a tool for mapping it can be used to support the development of educational qualifications programs across sites. Furthermore, the KLM should serve as guiding framework for every medical teacher.

The KLM delineates a profile of requirements for all teachers in medical education. It became clear during the consensus process that some positions and fields of duties might require additional competencies. The specific competencies will be defined and elaborated by our POiL working group in the near future.

## Acknowledgements

We thank all members of the GMA committee for personal and organizational development in teaching (POiL) for their contribution to the consensus process. We also like to thank the members of the network for medical education (MDN) for their support and advice and Johanna Feckl for proofreading this document.

## Notes

The position paper was accepted by the GMA executive board at 01-30-2015.

## Competing interests

The authors declare that they have no competing interests.

## Supplementary Material

The Core Competencies for Medical Teachers (KLM)

## Figures and Tables

**Table 1:**
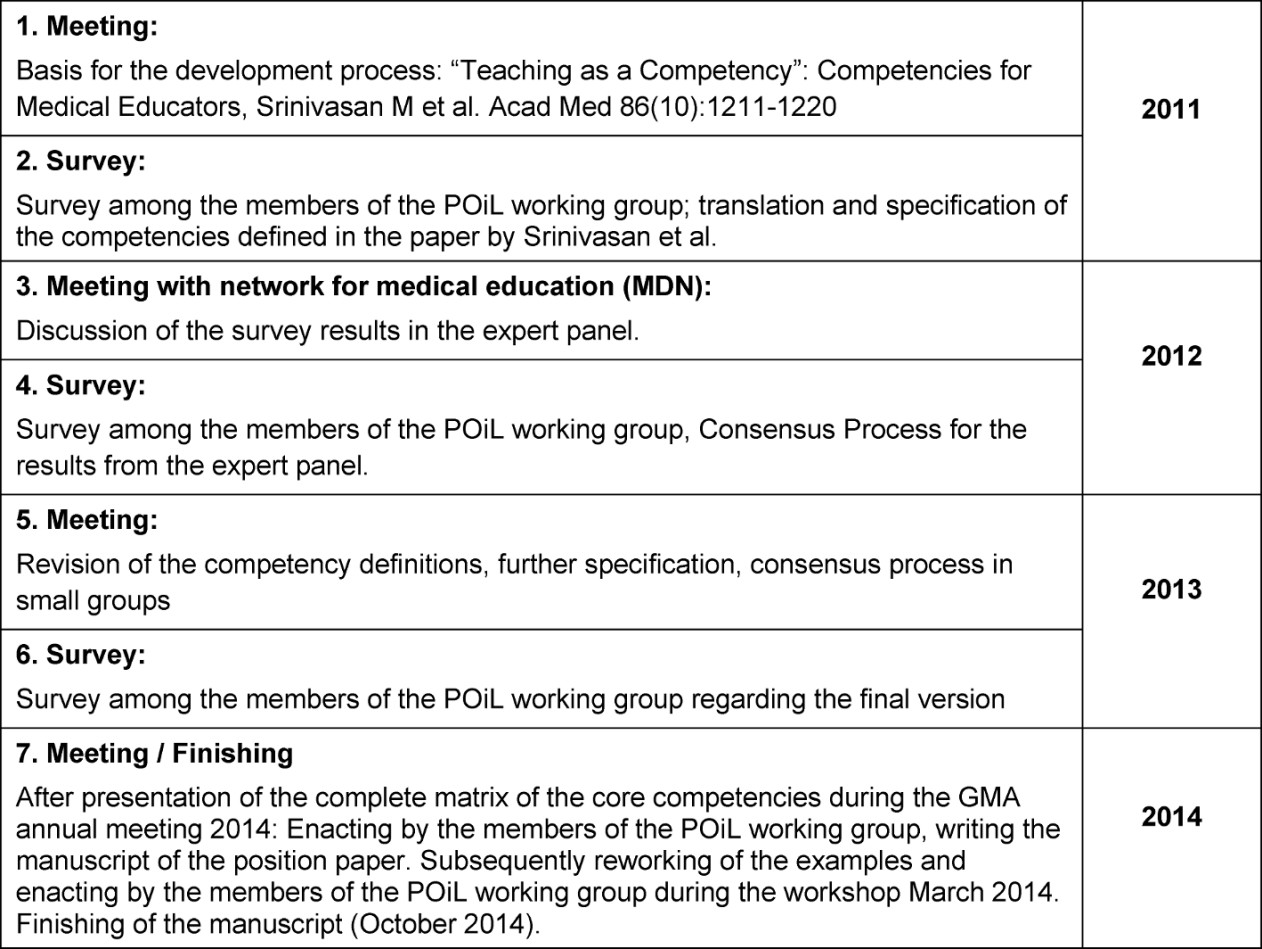
The development process

**Table 2:**
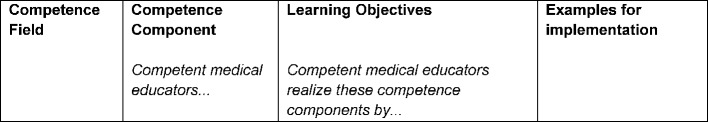
Structure of the competence model. A competence field embrace a number of competence components that are defined by specific learning objectives and respective examples for implementation.

**Table 3:**
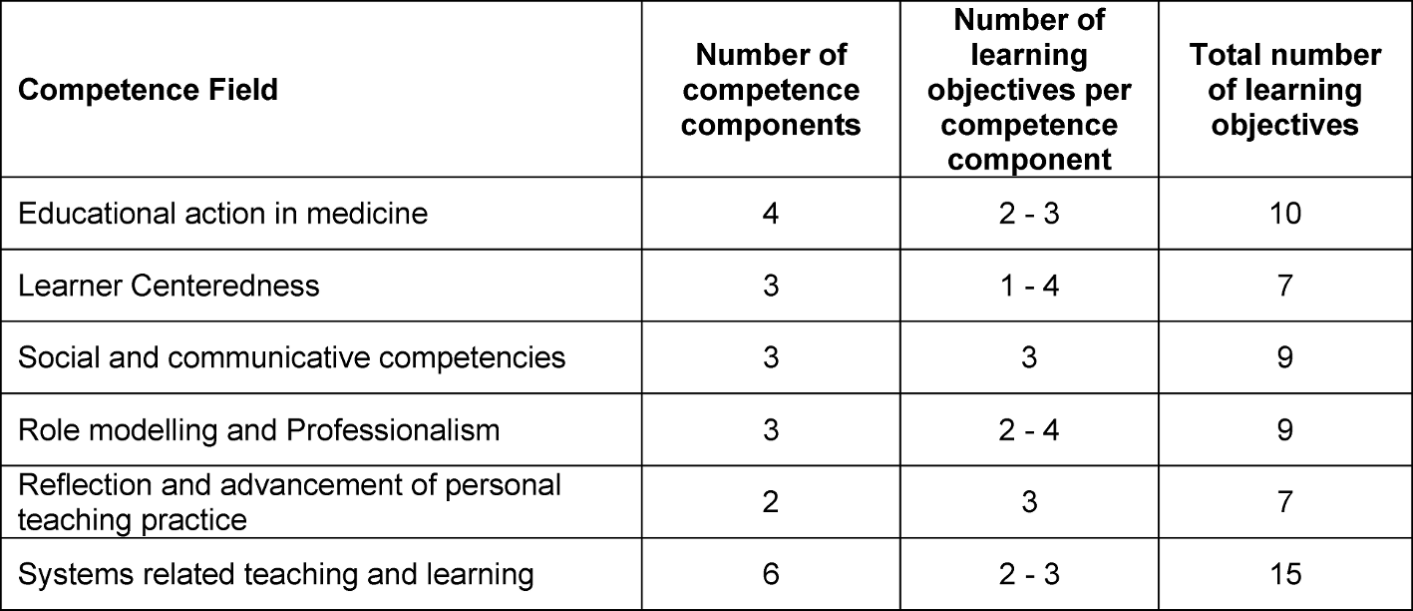
Number of competence components and learning objectives for each competence field of the KLM

**Table 4:**
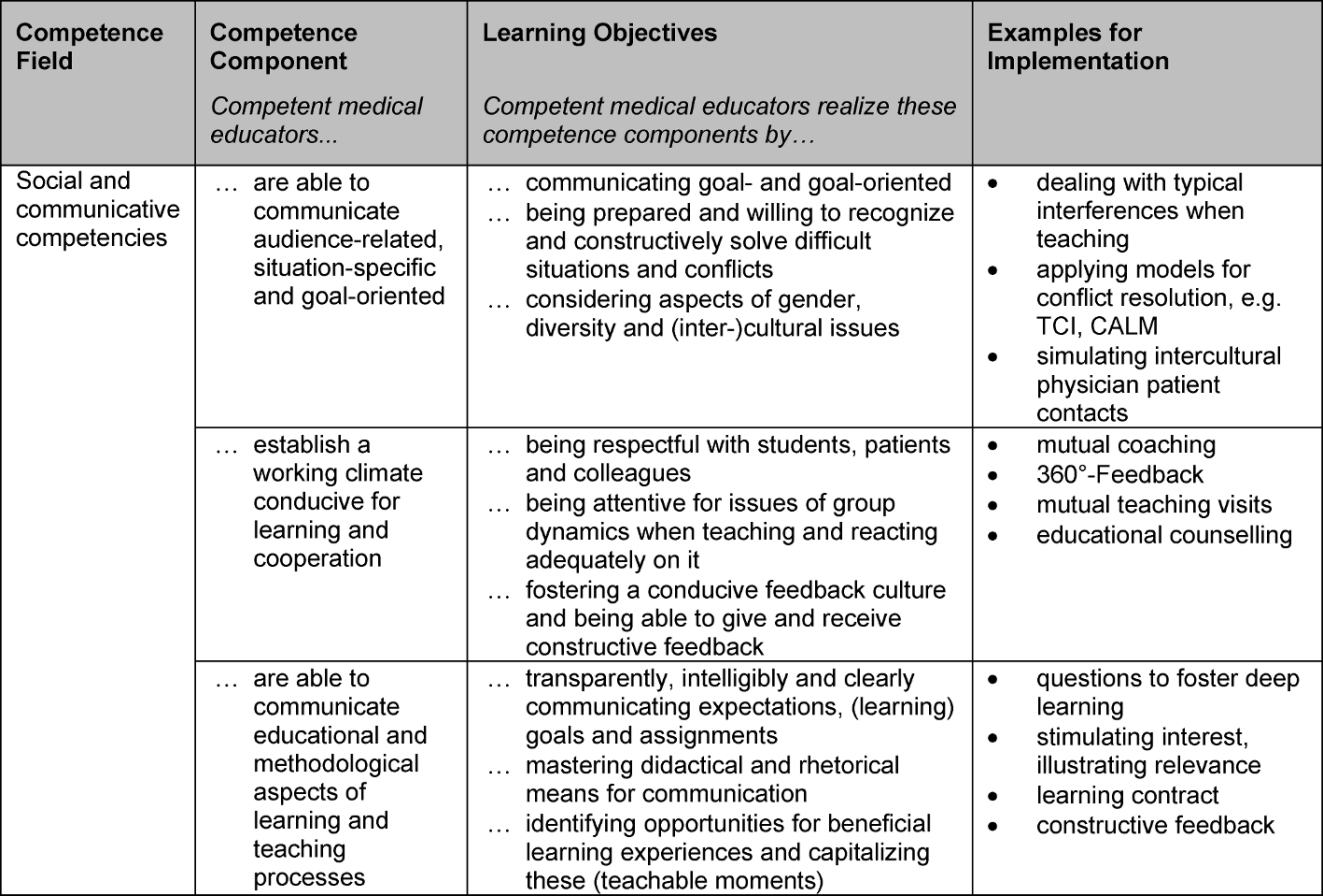
The competence field “social and communicative competencies” as a core competence for medical educators, divided into three competence components with corresponding examples for implementation
