# Supplementary material for: Core Competencies for Medical Teachers (KLM) – A Position Paper of the GMA Committee on Personal and Organizational Development in Teaching
Source: GMS Z Med Ausbild. 2015 May 13;32(2):Doc23. doi: 10.3205/zma000965 (PMC4446654; doi:10.3205/zma000965)
Supplement: The Core Competencies for Medical Teachers (KLM) [file ZMA-32-23-s-001.pdf]

**Competence Field**	**Competence Component**<br>*Competent medical educators...*	**Competence Field**<br>*Competent medical educators realize these competence components by..*	**Competence Component**
Educational action in medicine[1]<br><br>[1]*The necessary medical expertise is a precondition*	… are able to select appropriate subjects for instruction	… considering typical challenges and barriers for comprehension within their teaching domain and by targeting and tackling these with appropriate methods<br>… by boiling down the teaching content to what is necessary for understanding without simplifying unacceptably<br>… guiding students to adequately prioritize and apply the necessary knowledge and skills for health care in a target-oriented manner	• selecting subjects with regard to relevance, frequency, urgency and exemplarity<br>• e.g. indicating contraindications
	… are able to design conducive teaching and learning processes with regard to methodological and educational issues	… highlighting the relevance of the subject-specific content with regard to the interdisciplinary context of medicine<br>… considering evidence from learning psychology and physiology<br>… analysing and creating learning processes adequately with regard to the surrounding conditions and by applying suitable methods and media	• considering the age-group of students e.g. exam nerves or alcoholic intoxication during a party<br>• e.g. Guilbert's 5-points or SMART criteria for writing learning objectives<br>• Planning teaching by use of the Sandwich principle or the AVIVA model<br>• using social structures e.g. buzz groups or aquarium
	… adequately assess and evaluate the learning progress of their students with regard to knowledge, skills and attitudes	… using different assessment methods according to the situation<br>… aligning assessments with the curriculum by means of blueprints	• selecting assessment methods by means of Miller's pyramid or Bloom's taxonomy e.g. OSCE for assessment of skills<br>• to draft outcome expectancies
	… challenge students with demands that meet their previous knowledge and skills	… considering the general as well as the specific learning outcomes of the curriculum<br>… not demanding competencies from the students that are actual part of residency or PGME curricula	• e.g. not asking students to interpret a pacemaker ECG or letting students place a central line independently<br>• aligning the learning outcomes of one's own educational interventions with the defined outcomes of the curriculum

**Competence Field**	**Competence Component**<br>*Competent medical educators...*	**Competence Field**<br>*Competent medical educators realize these competence components by..*	**Competence Component**
Learner Orientation	… create an atmosphere conducive for learning	… appreciating students' contributions<br>… encouraging students to pose questions and express dissenting points of view<br>… showing evident interest regarding students' learning progress<br>… offering individual constructive feedback and highlighting individual room for improvement	• using methods for collaboration e.g. SNAPPS<br>• interactive teaching methods, e.g. group work, learning circles
	… support students' learning process allowing for their individual potential and needs	… recognizing learning difficulties and supporting students in overcoming these<br>… facilitating self-reflection also with regard to personal and emotional issues	• encouraging students to define goals, e.g. writing down goals during a clinical rotation<br>• suggesting students to critically reflect learning achievements, e.g. letting students write reflection reports
	… activate already existing prior knowledge	… respecting students' prior knowledge and motivation against the interdisciplinary context when planning and implementing different educational interventions	• Advance Organizer<br>• using methods to activate prior knowledge, e.g. quizzes, brainstorming, PBL
Social and communicative competencies	… are able to communicate audience-related, situation-specific and goal-oriented	… communicating goal- and goal-oriented<br>… being prepared and willing to recognize and constructively deal with difficult situations and conflicts<br>… considering aspects of gender, diversity and (inter-)cultural issues	• dealing with typical interferences when teaching<br>• applying models for conflict resolution, e.g. TCI, CALM<br>• simulating intercultural physician patient contacts
	… establish a working climate conducive for learning and cooperation	… being respectful with students, patients and colleagues<br>… being attentive for issues of group dynamics when teaching and reacting adequately on it<br>… fostering a conducive feedback culture and being able to give and receive constructive feedback	• mutual coaching<br>• 360°-Feedback<br>• mutual teaching visits<br>• educational counselling
	… are able to communicate educational and methodological aspects of learning and teaching processes	… transparently, intelligibly and clearly communicating expectations, (learning) goals and assignments<br>… mastering didactical and rhetorical means for communication<br>… identifying opportunities for beneficial learning experiences and capitalizing these (teachable moments)	• questions to foster deep learning<br>• stimulating interest, illustrating relevance<br>• learning contract<br>• constructive feedback

**Competence Field**	**Competence Component**<br>*Competent medical educators...*	**Competence Field**<br>*Competent medical educators realize these competence components by..*	**Competence Component**
Role modeling and Professionalism	… consider the congruence of educational objectives and their own professional conduct	… considering the physician's role and image as well as expectancies towards physicians and reflecting these aspects against the background of their own attitudes and behaviour<br>… considering ethical and legal norms and values and making these transparent whenever necessary<br>… considering the double role of physician and teacher when interacting with patients and students especially during clinical teaching	• considering ethical principles when teaching, e.g. using non-judgmental language<br>• sympathetically respecting patients' dignity, e.g. no stigmatization of patients<br>• highlighting decision structures and conflicts, e.g. transparently using meta-communication
	… convey the necessity for reflecting one's own attitudes and behaviour against the background of professional demands and expectations	… encouraging students to consider their own professional self-concept<br>… strengthening students' personal responsibility for the learning progress (-> Learner Orientation)<br>… adequately addressing unprofessional behaviour of students in class<br>… responsibly initiating appropriate actions to point out possible competence deficit to students and prompt them to initiate respective changes	• considering rules for hygiene, e.g. hand disinfection<br>• addressing inappropriate clothing of students e.g. flip-flops on the ward<br>• adequately assessing students' test performance e.g. letting students fail if appropriate<br>• supporting students, e.g. mentoring, feedback
	… are prepared to critically self-reflect and for lifelong learning	… analysing the strength and weaknesses to generate goals for their continuing professional development<br>… dealing openly with failures and using them for further development	• mutual coaching<br>• participating in training opportunities<br>• teaching portfolio<br>• critical incident method
Reflection and advancement of personal teaching practice	… reflect their own teaching practice	… using different forms of self-reflection<br>… using different forms of evaluation<br>… using scientific concepts and evidence<br>… aspiring to identify and accept own failures and searching for constructive solutions	• Reflective Writing<br>• 360° Feedback<br>• evaluation of students<br>• appraisal interviews<br>• reading up-to-date medical education literature

**Competence Field**	**Competence Component**<br>*Competent medical educators...*	**Competence Field**<br>*Competent medical educators realize these competence components by..*	**Competence Component**
	… continuously develop their own teaching practice	… using opportunities for further training in medical education<br>… defining goals for the development of their teaching competencies and verifying their accomplishments<br>… experimenting with different learning methods and materials	• CPD, e.g. participating in national and international trainings / workshops<br>• appraisal interviews<br>• considering the requirements / learning outcomes of certified programs for medical education<br>• broaden their variety of methods
Systems related teaching and learning	… utilize and create institutional conditions conducive for learning and teaching	… aligning the planned and assessed curriculum with their teaching content (constructive alignment)<br>… utilizing proprietary resources to improve conditions for learning and teaching	• blueprinting outcomes, learning content and methods, assessment formats and tasks<br>• utilizing the national catalogue for learning in medicine or similar documents<br>• organizing rooms that students can use for self-study<br>• computer labs for students<br>• providing learning materials online (learning platform)<br>• publish existing resources, e.g. simulation labs, training labs, learning platforms
	… consider the legal conditions for medical education	… knowing the relevant acts and regulations<br>… implementing the demands and requirements of these documents in their practice	• constitution, medical licensure act, local study and assessment regulations<br>• clarifying issues of liability e.g. when medical students act independently<br>• informing students about their rights and duties at the beginning of clerkships

**Competence Field**	**Competence Component**<br>*Competent medical educators...*	**Competence Field**<br>*Competent medical educators realize these competence components by..*	**Competence Component**
	… consider the structures and processes within their institution	… knowing the structures and responsibilities in their institution that are relevant for teaching<br>… considering the processes within their institution	• identifying the paths of information necessary for the coordination with the office of student affairs, program directors, etc. e.g. knowing the program director in a certain area
	… participate in the development of their school, faculty	… participating in the strategic development of the curriculum and contributing to the design of the local learning environment<br>… being willing to support and contribute to curricular innovations<br>… supporting and fostering the evaluation of teaching	• contributing to symposia, e.g. Teaching Day<br>• Dealing transparently with the results of the evaluation, e.g. publishing them for students
	… include societal and political developments when planning their teaching	… including foreseeable demographic developments<br>… considering aspects that are related to the health care system and the delivery of care<br>… considering developments in professional policy	• selecting cases specifically e.g. primary care for the elderly, inter-professional care
	… communicate with others to realize new aspects in teaching or to hand on their expertise to others	… actively seeking exchange with individuals outside of their institution<br>… handing on their experience and insights from their teaching projects<br>… being willing to cooperate beyond their department and school or faculty	• using platforms for scientific exchange, e.g. publishing teaching projects, presenting posters<br>• actively exchanging with other teachers e.g. by sharing teaching materials<br>• retrieving expertise from other sections of the university e.g. consultation for e-learning<br>• contributing to committees of the national organization for medical education.
